# Relationship between probability of future shoulder arthroplasty and outcomes of arthroscopic debridement in patients with advanced osteoarthritis of glenohumeral joint

**DOI:** 10.1186/s12891-015-0741-9

**Published:** 2015-10-05

**Authors:** Patrick Henry, Helen Razmjou, Tim Dwyer, Jesse A. Slade Shantz, Richard Holtby

**Affiliations:** Division of Orthopedic Surgery, Department of Surgery, Sunnybrook Health Sciences Centre, Toronto, ON Canada; Division of Orthopaedic Surgery, Department of Surgery, Faculty of Medicine, University of Toronto, Toronto, Canada; Holland Orthopaedic & Arthritic Centre, Sunnybrook Health Sciences Centre, 43 Wellesley Street East, Toronto, ON M1Y 1H1 Canada; Department of Physical Therapy, Faculty of Medicine, University of Toronto, Toronto, Canada; Sunnybrook Research Institute, Sunnybrook Health Sciences Centre, Toronto, Canada; Department of Orthopedic Surgery, Women’s College and Mount Sinai Hospital, Toronto, Canada; Orthopaedic Sports Medicine, Toronto, Canada; Department of Orthopaedic Surgery, Rouge Valley Health System, Toronto, Canada

**Keywords:** Debridement, Future arthroplasty

## Abstract

**Background:**

Arthroscopic glenohumeral debridement for symptom control has shown promising short term results in the young active population, when arthroplasty may not be a practical option due to the recommended activity restrictions, potential for complications and/or early wear, and a need for revision. The purpose of this study was twofold: 1) to examine the impact of arthroscopic debridement with or without subacromial decompression on clinical outcomes in patients with severe glenohumeral osteoarthritis (OA), and 2) to explore the differences in post-debridement outcomes between patients who eventually progressed to arthroplasty vs. those who did not. The role of an active worker’s compensation claim was examined.

**Methods:**

Prospectively collected data of patients who were not good candidates for shoulder arthroplasty and had subsequently undergone arthroscopic shoulder debridement were used for analysis. Disability was measured using the relative Constant-Murley score (CMS), the American Shoulder and Elbow Surgeon’s (ASES) assessment form, pain free range of motion (ROM), and strength.

**Results:**

Fifty-six patients were included in the final analysis. Eighteen (32 %) patients underwent arthroplasty surgery (arthroplasty group) over a period of 11 years. The arthroplasty group was comparable with the non-arthroplasty group prior to debridement but was more disabled at post-debridement surgery follow-up, functioning at less than 50 % of normal based on ASES, relative CMS, and active painfree ROM. In the multivariable analysis, the post-debridement relative CMS was affected by having a compensation claim and having a future arthroplasty.

**Conclusion:**

Arthroscopic debridement improved clinical outcome in 68 % of patients suffering from advanced OA of glenohumeral joint. Having less than 50 % of normal score in ASES, relative CMS and painfree ROM post- debridement within a period of two years may be an indication for future arthroplasty. Role of worker’s compensation claims should not be underestimated.

## Background

Glenohumeral osteoarthritis (OA) is a disabling condition characterized predominantly by pain, stiffness, and subsequent decreased functional activity. The impact of glenohumeral joint OA on quality of life is comparable to congestive heart failure, diabetes, and acute myocardial infarction [1]. Non-operative treatment may include lifestyle and activity modification, occupational modifications, rehabilitation, pain medication, and intra-articular corticosteroid injections.

For patients over the age of 60 who can comply with instructions to place relatively low functional demands on their shoulder, shoulder arthroplasty is the mainstay of treatment and provides reliable symptom relief [2]. However, in the young active population, arthroplasty may not be a practical option due to the recommended activity restrictions, potential for complications and/or early wear, and a need for revision [2–4].

Arthroscopic glenohumeral debridement for symptom control has shown promising short term results, especially in patients with subacromial osteophytes [2, 5–9]. Several variations of the procedure have been described, including arthroscopic debridement of loose chondral tissue, removal of loose bodies, capsulotomy, and microfracture, along with other concomitant arthroscopic procedures such as subacromial decompression, long-head of the biceps management, and distal clavicle resection [6, 10–16]. In most reports, these operative techniques have led to improved patient satisfaction and functional outcome scores. In many such studies however, the primary indication for surgery was not OA but other pathology, such as impingement syndrome, and the arthritic debridement was performed as a secondary procedure [10, 12–15]. Additionally, for many patients in these studies the arthritic process was so mild that it was not detected on pre-operative x-rays [6, 10, 13–15].

The purpose of this study was twofold: 1) to examine the impact of debridement with or without subacromial decompression on reported disability, painfree active range of motion and strength in patients with severe primary glenohumeral OA, and 2) to explore the differences in post-debridement outcomes between patients who eventually progressed to arthroplasty vs. those who did not. The role of an active shoulder related workers’ compensation claim was examined.

We hypothesized that on average all patients would show a significant improvement in their report of disability, strength and pain free range of motion after debridement, but that individuals who progress to arthroplasty would have higher levels of disability pre- and post debridement surgery.

## Methods

### Subjects

Existing data of patients with advanced primary OA of the glenohumeral joint who had undergone debridement from January 2001 to January 2012, and had one or two year follow-up were extracted from a surgical shoulder research database where prospectively collected data were stored. These patients were not considered ideal candidates for shoulder arthroplasty due to young age, high activity level, a desire to avoid major surgery at the time of assessment, or combinations thereof. Inclusion criterion was having advanced degenerative OA of the glenohumeral joint diagnosed on x-ray (joint space narrowing, inferior joint osteophytes), and confirmed as having Outerbridge Grade III/IV arthritic changes on the humeral head and glenoid at time of arthroscopy. Patients who had undergone any previous shoulder surgery, including a debridement or repair of the rotator cuff tear were excluded. Routinely, all patients completed a course of non-operative treatment for a minimum of six months prior to being considered for surgery.

Disability and functional outcome data at the 1 and/or 2 year post-operative mark were used for data analysis. In addition to reviewing data at the 1 and 2 year post-debridement time points, we searched the surgical database up to the date of data extraction (March 2015) to determine which patients either were progressed to or where booked for shoulder arthroplasty within that timeframe. This period varied from 2 to 11 years depending on the date of initial debridement surgery.

All patients had signed a written consent form and had provided permission for the information to be included anonymously for clinical research projects. Approval for using the existent database was obtained from the institutional Research Ethics Board of the Sunnybrook Health Sciences Centre (project ID# 085–2012).

### Surgical procedures

All surgical procedures were performed by the senior surgeon (RH), with the patient under general anesthesia in the lateral decubitus position. Arthroscopic debridement included glenohumeral joint synovectomy, as well as removal of loose bodies, chondral flaps and degenerative tissue. Resection of the distal end of the clavicle was performed when advanced OA was seen on preoperative Zanca radiographs, or when diagnosed by arthroscopy. Acromioplasty was performed in all patients with Bigliani type II or type III acromial morphology as diagnosed on a supraspinatus outlet view. Partial tears of biceps up to 50 % through the tendon were debrided, and tears more than 50 % were treated with tenodesis or tenotomy.

### Outcome measures

Limited and painful range of motion (ROM) is one of the most significant sequela of OA of the glenohumeral joint. Therefore, the relative Constant-Murley score (CMS) [17] which captures the impact of OA on ROM objectively was used as the primary outcome measure. Strength and pain free range of motion components of the relative CMS were analyzed separately. Pain free active ROM was assessed in four directions utilizing a score ranging from 0–40 (zero points for minimum, and ten points for maximum range in each direction). Using a tensiometer, strength was measured in pounds as the maximum force that the patient could resist on a single event for 5 s at the highest available elevation in the scapular plane. In addition, the American Shoulder and Elbow Surgeon’s (ASES) assessment form [18] was used as a subjective measure of disability. All measures were documented 2–3 weeks prior to surgery and at the follow-up visit (one or two years post-surgery). Both ASES and Constant Murley have established reliability and validity in patients with shoulder pathology [17–22]. Level of co-morbidity (0–52) was calculated as continuous data based on a validated score, the Cumulative Illness Rating Scale [23] which examines the overall health. In this scale, zero represents no impairment and 52 represents the highest level of possible impairment.

### Statistical analysis

Parametric and non-parametric analyses were used to examine the overall change in the entire sample and the difference in pre and post-operative scores and change over time in relative CMS, ASES, strength and pain free ROM between patients who progressed to arthroplasty and those who did not. Subgroup analyses examined the significance of change within each group. Analysis of Covariance (ANCOVA) examined the impact of future replacement on post-op relative CMS while adjusting for pre-op relative CMS. Multivariable analysis was performed to assess the difference in post-debridement scores of relative CMS between patients who progressed to arthroplasty and those who did not, while adjusting for pre-op relative CMS. Age and sex were not included in this analysis as relative CMS already adjusts for these differences. Statistical analysis was performed using SAS® version 9.1.3 (SAS® Institute, Cary, NC). Statistical results are reported using 2-tailed p values with significance set at *p* < 0.05.

## Results

The database contained 101 consecutive patients who had undergone debridement surgery over a period of 11 years (2001 to 2012). Twenty-eight patients (28 %) were missing both one and two year follow-up data and were excluded. Out of the remaining 73 patients, 17 patients had a concomitant rotator cuff tear and were excluded, leaving a total of 56 patients. There was no statistically significant difference (*p* > 0.05) in age, sex, symptom duration, pre-operative relative CMS, ASES, between excluded patients and those whose data were analyzed.

Table 1 shows demographics of the 56 patients (32 males, 24 females, average age = 59 ± 13) whose data were used for analysis. Twenty-eight (50 %) patients reported an insidious onset of symptoms, with the remaining 28 (50 %) recalling a causative event such as a fall, direct blow, or sports or work-related strenuous activities. Forty-seven patients (84 %) had either acromioplasty or resection of lateral clavicle. Ten (18 %) patients (three female, seven men) had an active workers’ compensation claim related to their shoulder and were significantly younger than the non-workers compensation group (mean age: 47 vs. 62, *p* = 0.001).

**Table 1:** Descriptive data of 56 patients

Variables	Number (%)
Age (years)	59, SD = 13 (25–82 y)
> = 60	29 (52 %)
<60	27 (48 %)
Sex	
Male	32 (57 %)
Female	24 (43 %)
Symptom duration (months)	84, SD = 92
Comorbidity (0–52)	3.50, SD = 3 (0–13)
Affected Side	
Left	14 (25 %)
Right	21 (37.5 %)
Bilateral	21 (37.5 %)
Side operated on	
Left	43 (24 %)
Right	32 (57 %)
Mechanism of Injury	
Insidious	28 (50 %)
Traumatic	28 (50 %)
Work-related injury	10 (18 %)
Night pain	33 (59 %)
Associated Surgeries	
Acromioplasty	46 (82 %)
Lateral clavicle resection	32 (57 %)
Biceps tenodesis	3 (5 %)
Biceps tenotomy	1 (2 %)
Loose body removal	1 (2 %)
*p* value of change in outcomes	
RCMS (0–110), *p* <0.0001	41 (18)/59(25)
ASES (0–100), *p* <0.0001	41 (18)/58(24)
Strength (0–30 lb), *p* =0.0002	6 (5)/9(6)
Painfree active ROM (0–40), *p* =0.0008	17 (10)/21(11)

All 56 patients had complete pre-operative data available. Twenty-seven patients had 1-year post-operative follow-up data available, 29 patients had 2-year follow-up data available, and 12 patients had both 1 and 2 year follow-up data available. There was no statistically significant difference between the 1 and 2 year post-operative functional outcome scores for the group of 12 patients that had this data available. Overall, improvement with surgery was statistically significant for relative CMS, ASES, strength (*p* < 0.0001) and active pain free ROM (*p* = 0.0008) on average for the entire sample.

### Progression to shoulder arthroplasty

Eighteen (32 %) of the 56 patients (11 women, seven men) had booked or had undergone shoulder arthroplasty surgeries at time of data extraction. The time frame between debridement and arthroplasty was a mean of 26 months (min: 13 months, max: 48 months). Only four (7 %) patients had follow-up of less than 48 months at the time of data extraction (had debridement after October 2008). The group of patients who ultimately underwent arthroplasty was similar to the no arthroplasty group with respect to age (mean age 63 vs. 58, *p* = 0.14), pre-operative symptom duration (91 vs. 80 months, *p* = 0.72), and sex [female 11/18(61 %) vs. male 7/18 (39 %)] (*p* = 0.057).

**Fig. 1:**
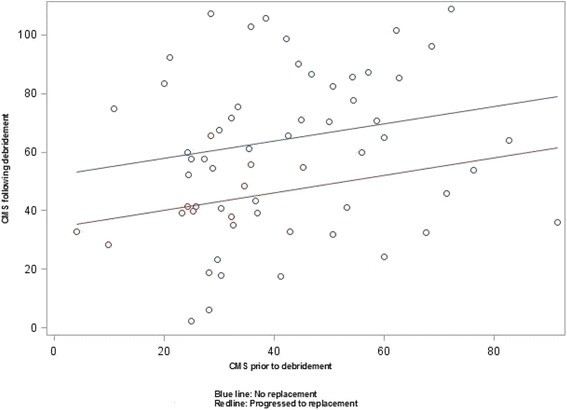
Post-debridement relative CMS in relation to pre-debridement scores in arthroplasty and no arthroplasty groups

Table 2 compares scores of relative CMS, ASES, strength and ROM prior to and following debridement surgery in the group of patients who progressed to arthroplasty with those who did not. Patients who progressed to joint arthroplasty during the study period were more disabled (based on relative CMS, ASES and pain free active ROM) at the 1 or 2 year post-debridement than those who did not progress to arthroplasty, despite being similar in relative CMS, ASES and ROM prior to their debridement procedure. The group difference in post relative CMS is shown in Fig. 1. The no-arthroplasty group demonstrated significant improvement when comparing pre- to post-operative scores, whereas the patients who ultimately progressed to arthroplasty did not experience the same benefit from the arthroscopic debridement procedure. In the arthroplasty group, the post-debridement scores of relative CMS and ASES remained below 50 % of the normal score of 100 with painfree ROM being below 50 % of the normal score of 40, whereas the no arthroplasty group passed the mean scores 50 %.

**Table 2:** Comparison between patients who progressed to arthroplasty with those who did not have arthroplasty within a period of 11 years

Variables	Arthroplasty (*N* = 18)	No Arthroplasty (*N* = 38)	Between group differences
RCMS (0–110)			
Pre debridement	38 (18)	43 (18)	NS
Post debridement	42 (18)	67 (25)	*P < 0.0001*
Change	5 (23)	24 (27)	*p = 0.01*
*P* values for change	NS	*P < 0.0001*	
ASES (0–100)			
Pre debridement	36 (18)	44 (18)	NS
Post debridement	42 (17)	65 (23)	*p = 0.0001*
Change	6 (20)	22 (29)	*p = 0.02*
*P* values for change	NS	*P < 0.0001*	
Strength (0–30 lbs)			
Pre debridement	4.8 (5.3)	4.9 (4.5)	NS
Post debridement	7.6 (5.5)	9.8 (6.5)	NS
Change	2.8 (7)	4.0 (6)	NS
*P* values for change	NS	*P = 0.0007*	
Painfree ROM (0–40)			
Pre debridement	14 (9)	18 (10)	NS
Post debridement	15 (8)	24 (11)	*p = 0.0006*
Change	0.11 (10)	6.9 (11)	*p = 0.03*
*P* values for change	NS	*P = 0.0004*	

*ASES* American Shoulder and Elbow Surgeon’s score

*RCMS* Relative Constant-Murley score

*ROM* Range of Motion

The ANCOVA showed a strong relationship (*p* = 0.0008) between future arthroplasty and post-debridement relative CMS scores (Fig. 1). In the multivariable analysis apart from future arthroplasty (*p* = 0.034), having a workers’ compensation claim (*p* = 0.0005) contributed to higher disability as measured by the relative CMS. In other words, the post-debridement score of relative CMS was strongly related to probability of heading towards future arthroplasty and having an active compensation claim.

## Discussion

The results of this study demonstrate that debridement for glenohumeral OA is an effective procedure to improve symptoms, pain free ROM and strength for the majority of patients (68 % of in our sample). However, at a mean of 26 months post-op, 32 % of the patients underwent or had booked joint arthroplasty. These patients experienced minimal improvement in symptoms following arthroscopic debridement, and remained below 50 % on their ASES and relative CMS scores. Apart from arthroplasty, post-operative scores of the relative CMS were affected by having a worker’s compensation claim - a factor that remains a strong predictor of recovery in patients with shoulder pathology.

Previous research reports on arthroscopic management of glenohumeral OA are relatively limited, such that the panel working on the American Academy of Orthopaedic Surgeons (AAOS) Clinical Practice Guideline on the Treatment of Glenohumeral Joint Osteoarthritis was unable to recommend for or against the use of arthroscopy as a management tool for arthritis, calling the available evidence “inconclusive” [24]. As is our report, all previous papers on the use of arthroscopy to manage OA in the shoulder are retrospective in design. However unlike our paper, most previous studies included patients whose primary diagnosis was not glenohumeral OA, and in many cases cartilage defects were only identified intra-operatively while the patient was undergoing arthroscopy for some other indication [6, 10, 13–15]. In our study however, the primary diagnosis and indication for surgery was the presence of radiographically advanced glenohumeral OA which we believe allows for more robust conclusions on the success or failure of the procedure.

In separate publications, Weinstein *et al.* [6], Cameron *et al.* [10] and Richards *et al.* [11] reported on 25, 61, and eight patients respectively, who had glenohumeral cartilage defects managed arthroscopically. In these studies, patients responded well overall to arthroscopic debridement, and in some cases a combination of lavage, debridement of cartilage flaps, removal of loose bodies, and various concomitant procedures depending on the types of intra-articular pathology encountered (*i.e.*, acromioplasty, distal clavicle excision, rotator cuff and/or SLAP tear debridement). Microfracture was not performed in any of these studies. Richards highlighted the importance of capsular release and its potential to improve pain by relieving pressure within the joint. The authors of all three of these studies agreed that patients with a greater severity of cartilage defects were less likely to benefit from the surgery. However, a similar study by Kerr concluded that the severity of arthritic defects did not predict outcome, but the presence of defects on both the humeral head and glenoid (“bipolar lesions”) did [13]. Again, glenohumeral OA was not the primary indication for surgery in most of these patients, leaving open a possible likelihood of selection bias.

Other studies have also evaluated the benefits of arthroscopy in so-called “pre-arthritic” stages of degenerative glenohumeral disease. Millet *et al.* [15] in 2009 and Frank *et al.* [14] in 2010 performed similar retrospective studies on 31 and 15 shoulders respectively, evaluating the benefits of arthroscopic debridement and microfracture of focal cartilage defects in the glenohumeral joint. As in the other studies, the cartilage lesions were not necessarily the primary indication for surgery, and were often encountered incidentally intra-operatively. Nonetheless, the techniques employed improved pain and ASES scores.

Apart from our current study, only one previous study has reported detailed results of arthroscopic management for the primary indication of degenerative OA of the shoulder. Van Thiel *et al.* [9] retrospectively analyzed and compared both pre- and post-operative outcome scores for patients who underwent arthroscopic management for a primary diagnosis of degenerative glenohumeral OA. Of the 71 patients who had adequate follow-up data, 16 patients (22 %) had undergone shoulder arthroplasty at a mean of 10.2 months after the arthroscopic procedure, and predictors of arthroplasty included the presence of grade 4 bipolar lesions, joint space of less than 2 mm, and large osteophytes. The remaining 55 patients experienced statistically significant improvements in active range-of-motion parameters, visual analog pain scores, ASES and SST scores, and had not had a subsequent shoulder surgery at a mean follow-up time of 27 months.

One of the main indications for arthroscopic management of arthritis within the glenohumeral joint is a desire to postpone or avoid arthroplasty. In our study, 18 of 56 patients (32 %) went on to arthroplasty at a mean 26 months (range 13 – 48 months) after the initial arthroscopic debridement. This is slightly higher than other studies, which have reported a rate of arthroplasty between 7 and 22 % over variable time periods following arthroscopic management of arthritic glenohumeral lesions [6, 10, 13–15]. However, the populations between studies may not be comparable, as our study included patients with symptomatic and radiographic evidence of advanced primary glenohumeral OA, while other studies included patients with no radiographic evidence of OA and very minor cartilage defects that were only found incidentally during arthroscopy indicated for some other pathology.

In summary, debridement for advanced primary glenohumeral OA improves symptoms, range of motion and strength for the majority of patients and this may help to delay surgery in patients who are not good candidates for arthroplasty for a variety of reasons. Patients who progressed to arthroplasty remained below 50 % on their ASES and relative CMS scores, which may be considered as an important indicator of a need for future arthroplasty.

### Limitations

The present study involved secondary analysis of patients whose data were prospectively collected. One limitation of our study is that the data for time of arthroplasty surgery was extracted at different time points for each patient. Although all patients in this study who eventually booked or underwent surgery did so within the maximum of 48 months, the rate of arthroplasty might be higher as four patients who had debridement procedure from 2008 to 2012 might eventually progress to arthroplasty. In addition, it is unknown what percentage of patients who were lost to follow-up went on to require arthroplasty.

## Conclusions

Arthroscopic debridement improved perceived disability, pain free active ROM and strength in 68 % of patients suffering from advanced OA of glenohumeral joint. Patients who progressed to shoulder arthroplasty on average had less than 50 % of normal score in ASES and relative CMS and painfree ROM post- debridement within a period of two years which may be used as a benchmark for future arthroplasty. Having an active worker’s compensation claim related to the shoulder was associated with an inferior outcome.

## Abbreviations

AAOS: American Academy of Orthopaedic Surgeons
ANCOVA: Analysis of Covariance
ASES: American Shoulder and Elbow Surgeon’s
CMS: Constant-Murley score
OA: Osteoarthritis
ROM: Range of motion
SD: Standard deviation
